# Supplementary material for: Intra-Tumoral Angiogenesis Is Associated with Inflammation, Immune Reaction and Metastatic Recurrence in Breast Cancer
Source: Int J Mol Sci. 2020 Sep 13;21(18):6708. doi: 10.3390/ijms21186708 (PMC7555442; doi:10.3390/ijms21186708)
Supplement: Supplementary file 1 [file ijms-21-06708-s001.pdf]

International Journal of *Molecular Sciences*

MDPI

Supplemental Materials

Masanori Oshi [1,2], Stephanie Newman[1,3], Yoshihisa Tokumaru [1,4], Li Yan [5], Ryusei Matsuyama [2], Itaru Endo [2], Masayuki Nagahashi [6] and Kazuaki Takabe [1,2,3,6,7,8,*]

1 Breast Surgery, Department of Surgical Oncology, Roswell Park Comprehensive Cancer Center, Buffalo, New York, NY 14263, USA; masa1101oshi@gmail.com (M.O.); snewman5@buffalo.edu (S.N.); Yoshihisa.Tokumaru@roswellpark.org (Y.T.); kazuaki.takabe@roswellpark.org (K.T.)
2 Department of Gastroenterological Surgery, Yokohama City University Graduate School of Medicine, Yokohama 236-0004, Japan. ryusei@yokohama-cu.ac.jp (R.M.); endoit@med.yokohama-cu.ac.jp (I.E.)
3 Department of Surgery, Jacobs School of Medicine and Biomedical Sciences, State University of New York, Buffalo, New York, NY 14263, USA.
4 Department of Surgical Oncology, Graduate School of Medicine, Gifu University, 1-1 Yanagido, Gifu 501-1194, Japan
5 Department of Biostatistics & Bioinformatics, Roswell Park Comprehensive Cancer Center, Buffalo, New York, NY 14263, USA; li.yan@roswellpark.org
6 Division of Digestive and General Surgery, Niigata University Graduate School of Medical and Dental Sciences, Niigata 951-8520, Japan; masanagahashi@gmail.com
7 Department of Breast Surgery, Fukushima Medical University School of Medicine, Fukushima 960-1295, Japan.
8 Department of Breast Surgery and Oncology, Tokyo Medical University, Tokyo 160-8402, Japan.
* Correspondence: kazuaki.takabe@roswellpark.org; Tel.: +1-716-8455540; Fax: +1-716-8451668

Received: 15 July 2020; Accepted: date; Published: date

Content

Table S1: Member genes of the Angiogenesis pathway score.
Figure S1: Histogram of angiogenesis score patient distribution in TCGA and METABRIC cohorts.
Figure S2: Association between the angiogenesis pathway score and survival of breast cancer patients in METABRIC cohort.

Table 1. Genes included in the Molecular Signatures Database (MSigDb) Hallmark angiogenesis gene set.

Gene Symbol	*Gene Name*
**APOH**	Apolipoprotein H
APP	Amyloid Beta Precursor Protein
CCND2	Cyclin D2
COL3A1	Collagen Type III Alpha 1 Chain
COL5A2	Collagen Type V Alpha 2 Chain
CXCL6	C-X-C Motif Chemokine Ligand 6
FGFR1	Fibroblast Growth Factor Receptor 1
FSTL1	Follistatin Like 1
ITGAV	Integrin Subunit Alpha V
JAG1	Jagged1
JAG2	Jagged2
KCNJ8	Potassium Inwardly Rectifying Channel Subfamily J Member 8
LPL	Lipoprotein Lipase
LRPAP1	LDL Receptor Related Protein Associated Protein 1
LUM	Lumican
MSX1	Msh Homeobox 1
NRP1	Neuropilin 1
OLR1	Oxidized Low Density Lipoprotein Receptor 1

PDGFA	Platelet Derived Growth Factor Subnit B
PF4	Platelet Factor4
PGLYRP1	Peptidoglycan Recognition Protein 1
POSTN	Periostin
PRG2	Proteoglycan 2
PTK2	Protein Tyrosine Kinase 2
S100A4	S100 Calcium Binding Protein A4
SERPINA5	Serpin Family A Member 5
SLCO2A1	Solute Carrier Organic Anion Transporter Family Member 2A1
SPP1	Secreted Phosphoprotein 1
STC1	Stanniocalcin-1
THBD	Thrombomodulin
TIMP1	TIMP Metallopeptidase inhibitor 1
TNFRSF21	Tumor Necrosis Factor Receptor Superfamily Member 21
VAV2	Vav Guanine Nucleotide Exchange Factor 2
VCAN	Versican
VEGFA	Vascular Endothelial Growth Factor A
VTN	Vitronectin

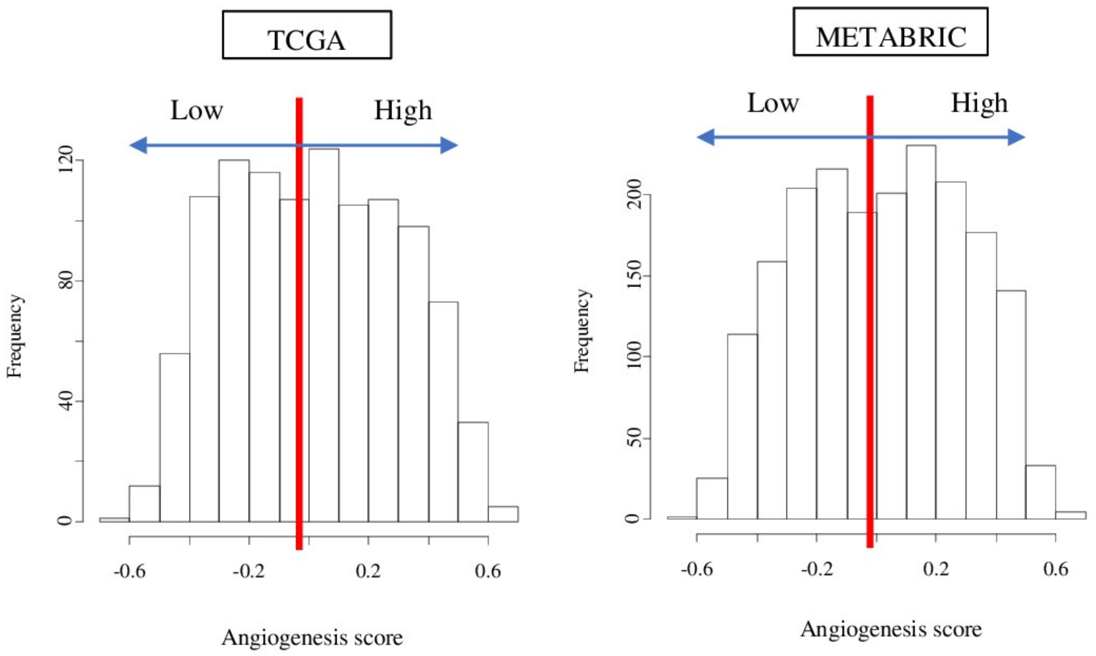

Figure 1. Histogram of angiogenesis score patient distribution in TCGA and METABRIC cohorts. Both cohorts demonstrate two weak peaks. Both cohorts were divided into High and Low score groups by the median value of the score (red line).

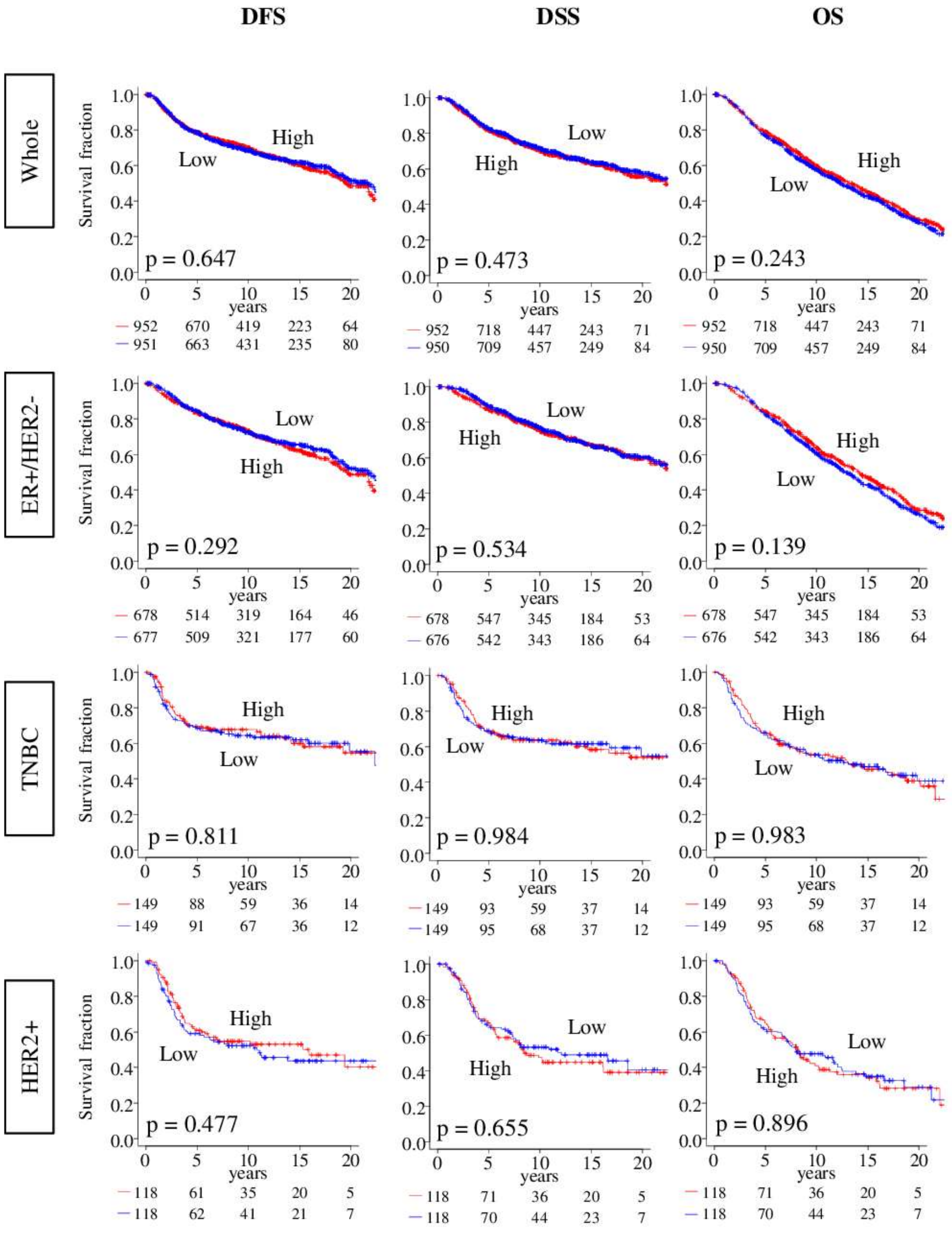

Figure 2. Association between the angiogenesis pathway score and survival of breast cancer patients in METABRIC cohort.
